# Preservation of Cadavers for Anatomic Studies

**Published:** 1899-11

**Authors:** 


### Preservation of Cadavers for Anatomic Studies.

The horrors and dangers of the dissecting room in the tropic climate of Rio Janeiro have been entirely obviated by Prof. Paes Leme's injections of formol, which preserves the tissues of a cadaver in a condition favorable for anatomic studies. A cadaver that has been kept twenty days in this way had the appearance of a fresh cadaver, in every respect, on the surface and when dissected. Anatomic pieces taken from these cadavers and kept in formol solution were in a perfect state of preservation, although in time they became mummified. One cadaver inspected had been preserved forty-eight hours by being wrapped in cloths moistened with the solution instead of injected. Tests are now in progress to determine whether the injections diminish the noxious effects of the cadaveric microbes and toxins as hoped and expected.—*Brazil Medico.*

